# All‐Polymer Solar Cells with 17% Efficiency Enabled by the “End‐Capped” Ternary Strategy

**DOI:** 10.1002/advs.202204030

**Published:** 2022-10-03

**Authors:** Yuchen Yue, Bing Zheng, Jianling Ni, Wenjie Yang, Lijun Huo, Jingxia Wang, Lei Jiang

**Affiliations:** ^1^ (CAS) Key Laboratory of Bioinspired Smart Interfacial Science Technical Institute of Physics and Chemistry Chinese Academy of Sciences Beijing 100190 P. R. China; ^2^ School of Chemistry Beihang University Beijing 100190 P. R. China; ^3^ School of Future Technology University of Chinese Academy of Sciences (UCAS) Beijing 100049 P. R. China; ^4^ Ji Hua Laboratory Foshan Guangdong 528000 P. R. China

**Keywords:** active layer morphology, all‐polymer solar cells, end‐capped, miscibility

## Abstract

Recently, all‐polymer solar cells (all‐PSCs) have received increasing attention and made tremendous progress. However, the power conversion efficiency (PCE) of all‐PSCs still lags behind the polymer‐donor‐small‐molecule‐acceptor based organic solar cells, owing to the excessive phase separation with poor miscibility between polymer donor and acceptor. In this research, an “end‐capped” ternary strategy is proposed by introducing PM6TPO as a third component to fabricate highly efficient all‐PSCs. The PM6:PM6TPO:PY‐IT based ternary devices exhibit impressive PCE of 17.0% with enhanced light absorption and optimal morphology, and the introduction of PM6TPO significantly reduces the phase separation. The ternary devices also exhibit improved stability, outstanding tolerance of active layer thickness, and high performance of 1 cm^2^ unit cells. More importantly, the “end‐capped” ternary strategy enables efficient and facile improvement of all‐PSCs performance without additional selection and complicated synthesis for the third component.

## Graphical abstract

A ternary strategy is proposed toward highly efficient all‐polymer solar cells (all‐PSCs) by introducing end‐capped PM6TPO. The champion device based on PM6:PM6TPO:PY‐IT exhibits an impressive power conversion efficiency (PCE) of 17.0%, which is attributable to the optimal morphology with improved miscibility and reduced phase separation. It is worth mentioning this is one of the highest PCEs known to the authors for all‐PSCs.

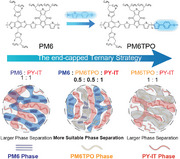

## 1 Introduction

Solution‐processed organic solar cells (OSCs) have received widespread attention, on the basis of their outstanding advantages in flexibility,^[^ ^1^, ^2^ ^]^ lightweight, and feasibility in large‐area production.^[^ ^3^, ^4^, ^5^ ^]^ In recent years, thanks to the innovation of the photovoltaic materials,^[^ ^6^, ^7^, ^8^, ^9^, ^10^ ^]^ interface engineering,^[^ ^11^, ^12^ ^]^ and deeper understanding of device physics,^[^ ^13^, ^14^, ^15^ ^]^ the power conversion efficiencies (PCEs) of polymer‐donor‐small‐molecule‐acceptor‐based OSCs have made an impressive progress of 19%.^[^ ^16^, ^17^ ^]^ Among various systems of OSCs, the all‐polymer solar cells (all‐PSCs) are considered as one of the most promising techniques for approaching application due to their improved devices stability^[^ ^18^, ^19^ ^]^ and film ductility.^[^ ^20^, ^21^ ^]^ Recent research efforts, especially the strategy of polymerized small molecular nonfullerene acceptors as polymer acceptor materials, have driven the PCEs of the all‐PSCs to exceed 16%.^[^ ^22^, ^23^, ^24^, ^25^, ^26^ ^]^ Nonetheless, the reported PCEs of all‐PSCs still lag behind polymer‐donor‐norfullerene‐acceptor based OSCs, largely due to the lack of further understanding and optimization of active layer morphology.^[^ ^27^, ^28^, ^29^, ^30^ ^]^ Typically, poor miscibility between polymer donor and acceptor leads to excessive phase separation, promoting the recombination of charge carrier and depressing the parameters of all‐PSCs performance.^[^ ^27^, ^31^, ^32^ ^]^

Ternary strategy is considered as one of the most efficient and facile approaches for the improvement of the OSCs performance by directly blending another donor/acceptor. In the classical ternary systems, these third components play a number of important roles, such as enhancement of light absorption, facilitation of charge transfer, and optimization of nanoscale morphology.^[^ ^33^, ^34^, ^35^, ^36^ ^]^ Reviewing recent research on ternary all‐PSCs, Liu and co‐workers have introduced BN‐T into the host binary system to fabricate the more crystalline active layer with improved exciton harvesting and charge transfer;^[^ ^37^ ^]^ Ma et al. used J71 as the third component, and obtained a PCE of 16.52% without damaging the initial film ductility;^[^ ^38^ ^]^ Li and co‐workers have introduced low‐cost PTQ10 as the second donor and obtained a higher efficiency with improved tolerance of active layer thickness.^[^ ^39^ ^]^ However, all the above third component need to be additionally synthesized, which undoubtedly increases the cost and complexity of further production. Moreover, achieving proper all‐polymer morphology with suitable phase separation is still a challenge.

In this research, we propose a novel ternary strategy based on the introduction of “end‐capped” polymer donor and achieve a high efficiency of 17.0%. The “end‐capped” strategy can also achieve the regulation of the aggregated state and miscibility, which are rarely reported in recent years. The PM6TPO, possessing additional end‐capped group with PM6,^[^ ^40^ ^]^ is introduced into previous proposed binary system PM6:PY‐IT as the third component. PM6TPO can be referred as “twins” polymer with PM6. Specially, the end‐capped ternary strategy avoids the complicated selection and additional synthesis for the third component. More importantly, the PM6TPO exhibits moderate aggregation and superior miscibility with the end‐capped group with steric effect, which shows excellent potential for achieving more suitable phase separation and optimal morphology. Through this strategy, an impressive 17.0% PCE of PM6:PM6TPO:PY‐IT based optimal ternary all‐PSCs is achieved (with the weight ratio of 0.5:0.5:1), which is higher than 15.49% and 15.26% for PM6 and PM6TPO based binary devices, respectively. The optimal ternary devices present a boosted short‐circuit current density (*J*_SC_) of 24.80 mA cm^−2^ (within 1% mismatch) and a higher fill factor (FF) of 72.52% without reduction of open‐circuit voltage (*V*_OC_) of 0.945 V, on the basis of highly efficient charge transport and enhanced light harvesting. The PM6:PM6TPO:PY‐IT based devices exhibit excellent storage stability and a decent tolerance in film thickness.

Then, further characterization demonstrates the ternary devices possess more efficient exciton dissociation, charge transfer, and more balanced charge transport. Furthermore, the morphology studies reveal that the introduction of PM6TPO can finely tune the molecular packing and induce more suitable phase separation length scale. This successful case not only gives a facile approach for highly‐efficiency all‐PSCs with one of the best performances among the all‐PSCs without additional selection of the third component, but also provides an effective solution for low‐cost morphology optimization.

## 2 Results and Discussion

**Figure 1:**
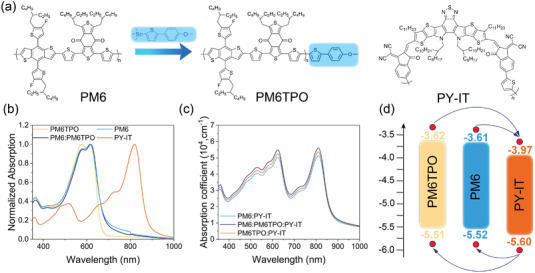
The chemical structure and properties of polymer donors and acceptor. a) The chemical structure of polymer donor PM6, end‐capped polymer donor PM6TPO, and polymer acceptor PY‐IT. b) The UV–vis absorption spectra of used materials. c) The UV–vis absorption spectra of PM6:PY‐IT, PM6TPO:PY‐IT, and PM6:PM6TPO:PY‐IT blend film. d) The energy level for PM6, PM6TPO, and PY‐IT.

The chemical structures of polymer donor PM6, polymer acceptor PY‐IT, and end‐capped polymer donor PM6TPO are shown in **Figure** **1**a (the molecular weight of polymer donor summarized in Table S1, Supporting Information). As shown in our previous report, the PM6TPO possessed additional end‐capped group with PM6 and can be synthesized by the stannylated end‐capped group to react with bromine‐terminated side of PM6 under Stille coupling conditions. The end‐capped polymer PM6TPO has the identical donor‐unit and acceptor‐unit to that of the commercial polymer PM6, hence PM6 and PM6TPO can be referred to as a set of “twin” polymers.

The normalized UV–vis absorption spectra of the materials in solid film (solution) are shown in Figure 1b; and Figure S1, Supporting Information, PM6 and PM6TPO exhibit absorption peak in 580 nm. Moreover, the PM6 has shown a stronger aggregated peak and redshifted *λ*_onset_. The PM6: PM6TPO binary blend film exhibits similar spectrum, with *λ*_onset_ between the PM6 and the PM6TPO based films (the detailed information collected in Table S2, Supporting Information). These results indicate the end‐capped PM6TPO exhibits a weaker crystallization and the blend films (PM6:PM6TPO) show moderate aggregation. As an acceptor material, the absorption spectrum of the PY‐IT neat film shows a significant peak of 820 nm, which is complementary to those of PM6 and PM6TPO. The binary and ternary blends absorption spectra are shown in Figure 1c, the ternary blend film has significantly enhanced absorption coefficient ranging from 500 to 850 nm, which is mainly due to the optimization of film morphology and general aggregation of polymer donor and acceptor. The energy level of material used in the active layer is presented in Figure 1d and the detailed optical properties, HOMO and LUMO are presented in Table S2, Supporting Information. The PM6 and PM6TPO present the similar HOMO (LUMO) of −5.52 (−3.61) e*V* and −5.51 (−3.62) eV, respectively, which are comparable with previous literatures. The HOMO and LUMO levels are −5.60 and −3.97 eV for acceptor PY‐IT, which are well matched with these polymer donors.

**Figure 2:**
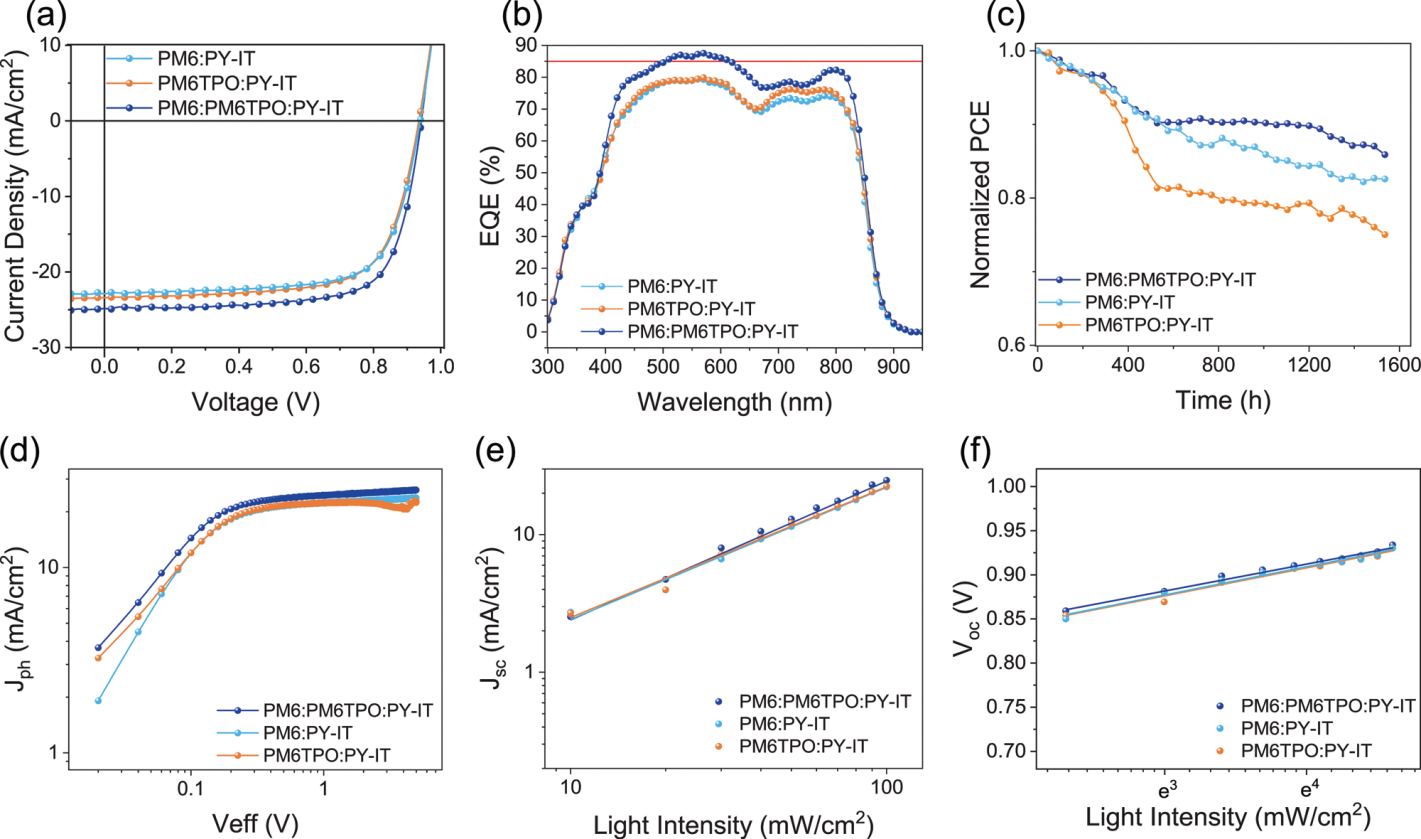
The characterization of the binary and ternary devices. a) The *J*–*V* curves of binary and ternary all‐PSCs under simulated AM 1.5 G illumination at 100 mw cm^−2^. b) The EQE spectra of the corresponding devices. c) The storage stability of the binary and ternary devices. d) The *J*_ph_ versus *V*_eff_ of the binary and ternary all‐PSCs. e,f) Light intensity dependence of *J*_SC_ and *V*_OC_ values of the corresponding OSCs.

To further investigate the photovoltaic performance of the binary and ternary devices, all‐PSCs were fabricated on the basis of a conventional device structure of ITO/PEDOT:PSS/active layer/PNDIT‐F3N/Ag. For all the devices, the donor: acceptor (D:A) weight ratio is 1:1, and the detailed fabrication processes are summarized in the Supporting Information. The current density–voltage (*J*–*V*) curves are exhibited in **Figure** **2**a, and corresponding detailed device parameters are summarized in **Table** **1**. The PM6:PY‐IT yields a PCE of 15.49% with a *J*_SC_ of 22.96 mA cm^−2^, a *V*_OC_ of 0.940 V and an FF of 71.77%. In comparison, the PM6TPO:PY‐IT based all‐PSCs achieve similar photovoltaic parameters with a PCE of 15.26%, a *J*_SC_ of 23.43 mA cm^−2^, a *V*_OC_ of 0.937 V, and an FF of 69.50%. Due to the similar properties of PM6 and PM6TPO, these binary systems possess similar photovoltaic performance, and the PM6TPO:PY‐IT based all‐PSCs exhibit a relatively lower FF and higher *J*_SC_ due to subtle differences in the aggregation state of these two “twins” polymers.

**Table 1:** Summarized photovoltaic parameters of binary and ternary all‐PSCs

*J*_SC_ [mA cm^−2^]	*V*_OC_ [V]	FF [%]	PCE [%]	PM6:PM6TPO:PY‐IT
22.96^a)^/22.31^b)^	0.940^a)^	71.77^a)^	15.49^a)^/(15.22±0.25)^c)^	1:0:1
24.80^a)^/24.60^b)^	0.945^a)^	72.52^a)^	17.0^a)^/(16.73±0.3)^c)^	0.5:0.5:1
23.43^a)^/22.72^b)^	0.937^a)^	69.50^a)^	15.26^a)^/(15.03±0.23)^c)^	0:1:1

^a)^ The best performance of devices;

^b)^ The integrated *J*_SC_ values from EQE spectra;

^c)^ Values in brackets are average based on more than 30 independent devices.

By the introduction of PM6TPO into PM6:PY‐IT classical binary system, we found that *J*_SC_ and PCE obviously increased. While the ternary all‐PSCs with the weight ratio of 0.5:0.5:1 for PM6:PM6TPO:PY‐IT yield much higher photovoltaic performance than those of binary systems. For the best device of ternary all‐PSCs, an impressive PCE of 17.0% with a *J*_SC_ of 24.80 mA cm^−2^, a *V*_OC_ of 0.945 V and an FF of 72.52% is recorded, which is one of the best performances of the all‐PSCs. The photovoltaic parameters with the different weight ratios of PM6TPO are collected in Figure S2 and Table S3, Supporting Information. The external quantum efficiency (EQE) spectra of all‐PSCs are presented in Figure 2b, and the integrated photocurrent from the EQE spectra matches well with the *J*_SC_s measured from the *J*–*V* curves (for optimal ternary devices, within 1% mismatch). The spectra are obviously enhanced from 400 to 850 nm by the introduction of PM6TPO, which is consistent with the above UV–vis absorption spectra and leads to the increased *J*_SC_s. Moreover, the storage stability of binary and ternary devices was investigated under the dark condition in nitrogen atmosphere. The PM6:PM6TPO:PY‐IT based all‐PSCs present the best performance in this research, the ternary devices maintain more than 90% of the initial performance over 1200 h, and more than 85% over 1600 h. The photo stability of all the devices was measured, and the ternary devices also exhibited the best performance, which maintains an initial efficiency of 85% after more than 400 h. Additionally, we have measured the thermal stability for ternary and binary devices, which were heated at 100 °C in the nitrogen atmosphere. The PM6:PM6TPO:PY‐IT based ternary devices exhibited the best performance, which still remains an initial efficiency of 85% after 96 h of heating (shown in Figure S3, Supporting Information).

Furthermore, the charge mobility of neat films has been also measured, and the results are illustrated in the Figure S4 and Table S4, Supporting Information. The PM6 and PM6TPO exhibit the hole mobility of 1.34 × 10^−3^ and 1.23 × 10^−3^ cm^2^ V^−1^ s^−1^, respectively. The neat film of PY‐IT shows the electron mobility of 1.10 × 10^−3^ cm^2^ V^−1^ s^−1^. Moreover, we conducted the characterization of hole and electron mobility (*µ*_h_ and *µ*_e_) of the binary and ternary blends by the space charge limits current (SCLC) method (shown in Table S4 and Figure S5, Supporting Information). For PM6:PY‐IT and PM6TPO:PY‐IT binary systems, the *µ*_h_:*µ*_e_ are 1.38 and 1.27, respectively. Compared to the two binary blends, the ternary devices exhibited the most balanced *µ*_h_:*µ*_e_ of 1.21, which is benefitted from well‐restrained bimolecular recombination and enhancement of FF.

To reveal the origin of the improved performance of the ternary all‐PSCs, the dependency of photocurrent (*J*_ph_) versus effective voltage (*V*_eff_) was measured (shown in Figure 2d, and corresponding parameters are summarized in Table S5, Supporting Information). The *J*_ph_ is given as *J*_ph_ = *J*_L_‐*J*_D_, *J*_L_, and *J*_D_ are defined as current density under illumination and dark condition, respectively, and the *J*_max_ is defined as current density on maximal output point. The *V*_eff_ is defined as *V*_eff_ = *V*_0_‐*V*_app_, the *V*_0_ is the voltage when *J*_ph_ = 0 mA cm^−2^ and the *V*_app_ is the applied voltage. The *J*_sat_ can be defined as *J*_ph_s reaching saturated state at *V*_app_ = 2 *V*. As shown in Figure 2d, the efficiencies of charge dissociation (*η*_diss_) and charge collection (*η*_coll_) can be calculated by the formulas of *J*_ph_ */J*_sat_ and *J*_max_ */J*_sat_, the ternary all‐PSCs present the maximal *η*_diss_ and *η*_coll_ of 97.58% and 85.58%, respectively. The above results indicate that the introduction of the end‐capped PM6TPO facilitates the exciton dissociation and charge collection.

To further investigate the charge recombination kinetics of the binary and ternary all‐PSCs, the dependency of *J*_SC_ and *V*_OC_ versus light intensity is measured.^[^ ^41^, ^42^ ^]^ As shown in Figure 2e, the fitting slope of the log (*J*_SC_) versus log (*P*_light intensity_) are 0.990, 0.966, and 0.960 for ternary devices, PM6 and PM6TPO based binary systems, respectively. These results indicate that the PM6:PM6TPO:PY‐IT based devices possess less charge recombination. Furthermore, the relationship of *V*_oc_ versus In (*P*_light intensity_) is defined as $V_{\mathrm{oc}}\propto n\times \left(\frac{\mathrm{KT}}{q}\right)/\mathrm{In}\;P_{\mathrm{light}\;\mathrm{intensity}}$ in Figure 2f, where *K* is Boltzmann's constant, *T* is temperature, and *q* is the elementary charge. The *n* values are 1.16, 1.24, and 1.25 for ternary devices, PM6 and PM6TPO based binary devices, respectively. The ternary devices exhibit the least trap‐assisted recombination and monomolecular recombination. The above results indicate that PM6:PM6TPO:PY‐IT based all‐PSCs possess the more efficient charge transport and higher FF.

**Figure 3:**
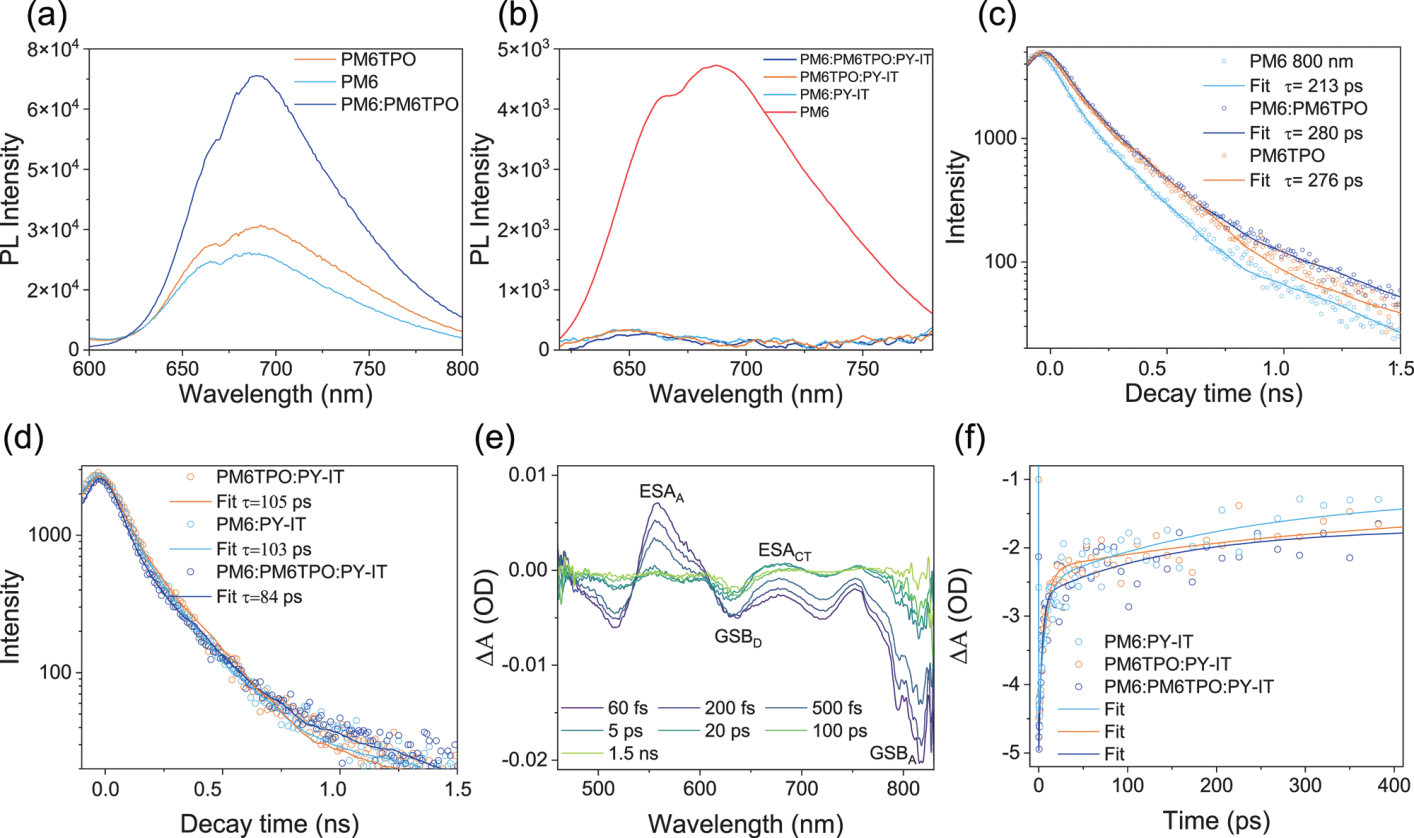
The measurement of charge transfer. a) The PL spectra of the materials used in this research. b) The PL spectra for the PM6:PY‐IT, PM6TPO:PY‐IT, and PM6:PM6TPO:PYIT blend film. C) TRPL for PM6, PM6TPO, and PM6:PM6TPO film. D) TRPL for corresponding binary and ternary blend film. E) The fs‐TA spectra recorded ranging from 60 fs to 1.5 ns for PM6:PM6TPO:PY‐IT. F) The fitting curves at GSB_D_ for PM6:PY‐IT, PM6TPO:PY‐IT, and PM6:PM6TPO:PT‐IT.

To further study the effect of the end‐capped PM6TPO as the third component on all‐PSCs performance, the steady‐state photoluminescence (PL) spectra and time‐resolved photoluminescence (TRPL) spectra were conducted to analyze charge generation and transport dynamic. The PL spectrum of PY‐IT neat film is collected in Figure S6a, Supporting Information, and the PY‐IT presents a broad emission peak ranging from 800 to 1000 nm. As shown in **Figure** **3**a, the PL spectra of PM6:PM6TPO blend film together with those of PM6 and PM6TPO neat films were measured at excitation wavelength of 600 nm, all the films exhibit the emission peak at 700 nm. The PM6:PM6TPO binary film presents the much higher PL intensity, indicating the efficient energy transfer between the “twins” polymer. After blending with polymer acceptor PY‐IT (shown in Figure 3b), the PM6:PM6TPO:PT‐IT based film exhibit the most PL quenching compared with these two binary films, and all the PL peaks blue shift to 650 nm, which indicates that the ternary films show the most efficient charge transfer between “twins” polymers donor and polymer acceptor.

Next, the TRPL spectra were used to further study the charge transfer with the introduction of end‐capped PM6TPO. The TRPL spectra of donor‐only films are shown in Figure 3c, the decay time was measured at 800 nm with the emission laser wavelength of 400 nm according to the above PL spectra, the PM6:PM6TPO binary film exhibit a relatively longer lifetime and larger PL quantum yield (PLQY) compared with these two neat films (shown in Figure S7, Supporting Information). These results imply that mixing of PM6 and PM6TPO can reduce nonradiative recombination. The TRPL spectrum for PY‐IT based neat film is presented in Figure S6b (Supporting Information), and *τ* = 172 ps. As shown in Figure 3d, the decay time at 800 nm was shortened after blending with the polymer acceptor PY‐IT, and the ternary films show a relatively shorter lifetime of 84 ps (achieved at the optimal ternary blends), while the PM6 and PM6TPO based binary films exhibit 103 and 105 ps, respectively. The above results are consistent with the enhanced EQE spectra of ternary all‐PSCs, especially from 600 to 850 nm.

The femtoseconds‐transient absorption spectra (fs‐TA) were selected to gain deep insight of the excitons generation and dissociation dynamics for the PM6:PM6TPO:PY‐IT ternary films and these two binary films.^[^ ^43^, ^44^ ^]^ The pump laser was set at 820 nm for selectively exciting acceptor, and spectra recorded at different decay time are shown in Figure 3e. There is an evident ground state bleach (GSB) recorded at 810 nm, which is resulted in the PY‐IT excited state, and a broad excited state absorption (ESA) of acceptor was observed at 560 nm. The intensity of GSB (donor at 630 nm) increased rapidly and started to decay slowly, which suggested that the charge transfer (CT) state was generated by exciton diffusion after the ultrafast hole transfer at the D/A interface, and then recombination to ground state. The kinetic trace at 630 nm (GSB for donor) for ternary and binary films is collected and compared in Figure 3f, the ternary blend film gives the shortest charge transfer lifetime, which exhibits that the introduction of PM6TPO can facilitate the CT process. The faster CT process in the PM6:PM6TPO blend may lead to higher photocurrent in the all‐PSCs, which is consistent with the improved performance of the ternary all‐PSCs.

**Figure 4:**
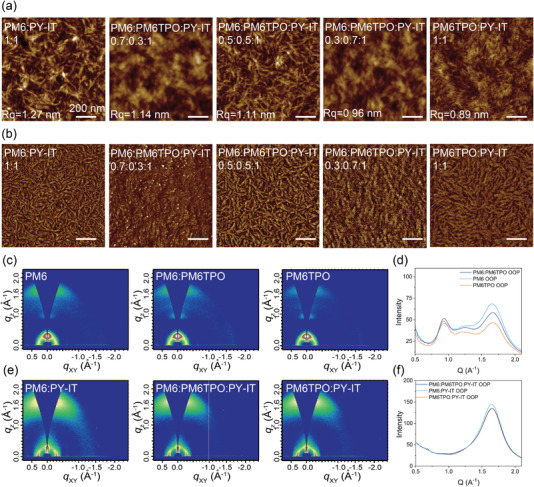
The measurement of the binary and ternary films morphology. a,b) AFM height and phase images of the binary and ternary blend film with different content. c) GIWAXS patterns of the PM6, PM6TPO, and PM6:PM6TPO film. d) OOP Line cuts of GIWAXS of the neat films. e) GIWAXS patterns of the binary and ternary blend film. f) OOP Line cuts for corresponding GIWAXS patterns of the binary and ternary blend film.

The active layer morphology plays an important role in the understanding of the photovoltaic performance and working mechanism, the atomic force microscopy (AFM) was used to confirm the binary and ternary film morphology. The AFM height and phased images are collected in **Figure** **4**a,b, and corresponding AFM images of neat film are shown in Figure S8, Supporting Information. The donor‐only films exhibit gradually decreasing root‐mean‐square roughness from 0.86 nm of PM6 neat film to 0.74 nm of PM6TPO neat film with the introduction of PM6TPO. The PY‐IT pristine film exhibit an Rq of 1.54 nm, which is shown in Figure S9a, Supporting Information. There are clear nanofiber interpenetrating network structures in all the binary and ternary blends, which is proved to be beneficial for efficient charge generation and dissociation. Compared with the relatively rougher surface and larger phase separation of PM6:PY‐IT based binary film (Rq = 1.27 nm), the roughness of the whole system decreases continuously with the introduction of PM6TPO. The PM6:PM6TPO:PY‐IT (0.5:0.5:1) blend shows a relatively decreased Rq of 1.11 nm, the PM6TPO:PY‐IT based blend exhibits the smallest Rq of 0.89 in these blends. The above results proved that PM6TPO can effectively reduce the crystallinity of the donor phase, thereby achieving the enhancement of miscibility and possession of balanced charge transport.

The molecular packing and crystalline ordering of neat and blend film were characterized by grazing incident wide angle X‐ray scattering (GIWAXS).^[^ ^45^ ^]^ As illustrated in Figure 4c, all the “twins” PM6 and PM6TPO and their binary films (with the weight ratio of 1:1) possess pronounced scattering peaks (010) in the out‐of‐plane (OOP) and highly ordered lamellar stacking peaks (100) in the in‐plane direction. The line cuts of IP direction are illustrated in Figure S10, Supporting Information. The line cuts of OOP direction are shown in Figure 4d and all the detailed parameters are summarized in Table S6, Supporting Information. With the combination of GIWAXS pattern and line cuts, all these films possess the *π*–*π* stacking diffraction peaks at ≈1.66 Å^−1^ from the line‐cuts of OOP direction, and the diffraction peaks located at 0.293 Å^−1^ in the IP orientation are also observed. Moreover, the crystalline coherence length (CCL) can be calculated according to the Scherrer equation, and CCLs of the PM6, PM6TPO, and their binary films are 21.26, 19.98, and 18.84 Å, respectively. The above results indicated that the introduction of end‐capped group could appropriately decrease aggregation of PM6, and the blending of these “twins” polymer can effectively achieve fine control of crystallinity. The PY‐IT neat film exhibit pronounced scattering peaks (010) in the OOP direction with a CCL of 22 .50 Å (presented in Figure S9b,c, Supporting Information). When blending with polymer acceptor PY‐IT, these binary and ternary films exhibited similar scattering peaks in the OOP direction, and corresponding *π*–*π* stacking diffraction peaks at ≈1.65 Å^−1^ (the distance of 3.8 Å), which can be seen in Figure 4e,f and the detailed parameters are collected in the Table S6, Supporting Information. The CCLs of PM6:PY‐IT, PM6:PM6TPO:PY‐IT, and PM6TPO:PY‐IT based films are 16.90, 16.80, and 16.70 Å in the OOP direction, respectively, which suggested that the “end‐capped” PM6TPO shows better miscibility with PY‐IT. The ternary film maintains the crystallinity and miscibility between the two binary films, which revealed that blending the PM6 and PM6TPO allows precise control of morphology and facilitates more efficient charge generation and transport.

The miscibility between the materials can be speculated by the Flory–Huggins interaction parameter *χ*_Α‐Β_, which is inferred from the surface tension (*γ*) of the materials via the equation of $\chi \propto {\left(\sqrt{\gamma_{A}}-\sqrt{\gamma_{B}}\right)}^{2}$,^[^ ^46^, ^47^ ^]^ and the *γ* of neat films were measured from the contact angle with water and glycerol by the Wu's model.^[^ ^48^ ^]^ All the contact angles are shown in Figure S11 (Supporting Information), and the detail *γ* and *χ*_Α‐Β_ are summarized in Tables S7 and S8 (Supporting Information). The PM6, PM6TPO, and PY‐IT possess the *γ* of 19.82, 20.25, and 23.11 mN m^−1^, respectively. These results indicated that PM6TPO has excellent miscibility with PM6 (the value of *χ* is 0.0023), and PM6TPO shows better miscible with PY‐IT than PM6, which demonstrated PM6TPO can improve the miscibility between donor and acceptor phase.

**Figure 5:**
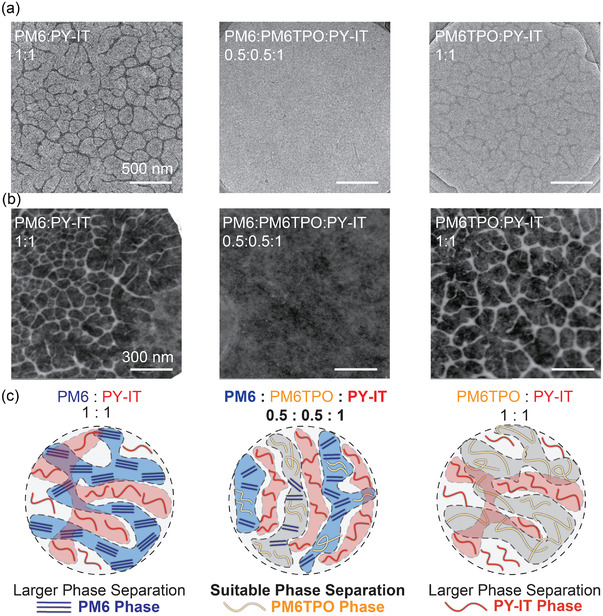
The morphology evolution mechanism of the end‐capped third component introduction. a,b) TEM and DF‐STEM images of the binary and ternary blend film with different content. c) Schematic illustration of the morphology evolution mechanism. Both the binary films exhibit larger phase separation, but the ternary blend show significant more suitable phase separation.

The working principle of ternary OSCs is closely dependent on morphology of blend film. Therefore, the transmission electron microscope (TEM) and dark‐field transmission electron microscope (DF‐STEM) were used for visual characterization of the phase separation and morphology. The DF‐STEM are imaged by scattered electrons, and the bright area represents crystalline domains. As shown in **Figure** **5**a,b; and Figure S12 (Supporting Information), the DF‐STEM provides a clearer morphology evolution process with different PM6TPO content. The DF‐TEM were imaged by scattered electron of sample, which is considered to be an effective method to identify the distribution of crystalline domain. These two binary blends show similar thick fibrillar structure in film, which represent both the binary blend possess large phase separation length scale with “over‐aggregated” crystalline phase. The optimal ternary blend films (0.5:0.5:1) exhibit exceptional finer and more evenly distributed fibers, which indicated the large‐content introduction of PM6TPO greatly affects the size and distribution of the crystalline domain. These results indicate that the optimal ternary blend possess more suitable phase separation (30–40 nm), which resulted in the efficient exciton harvesting and dissociation, and the morphology evolution process of the “twins” ternary system are schematically illustrated in Figure 5c.

For further industrial production of the all‐PSCs, the photovoltaic performance of devices should possess outstanding tolerance of the active layer thickness. For this reason, we fabricated PM6:PM6TPO:PY‐IT based ternary all‐PSCs with different active layer thicknesses ranging from 80 to 200 nm. The corresponding results are exhibited in Figure S13 and Table S9, Supporting Information. The *V*_OC_ of devices decreases slightly from 0.950 to 0.934 V with the increasing of active layer thickness, and the values of FF possess a similar decreasing trend, which mightily can be ascribed to the increased charge recombination and film resistance. The values of *J*_SC_ continuously increased with active layer thickness increasing from 80 to160 nm, and then decreased with further increasing of the thickness, the increasing of *J*_SC_ probably is due to the enhancement of light absorption. The highest PCE of 17.0% is achieved with the active layer thickness of ≈140 nm. Moreover, the PM6:PM6TPO:PY‐IT based ternary all‐PSCs exhibited excellent tolerance of the active layer thickness, the devices possess decent efficiencies >16% with thickness ranging from 80 to 160 nm. A high PCE of 14.62% is obtained for the all‐PSCs with the thickness of 200 nm. Meanwhile, 1 cm^2^ all‐PSCs are considered as the important unit cell for large‐area photovoltaic module. Hence, 1 cm^2^ all‐PSCs are fabricated based on the PM6:PM6TPO:PY‐IT, which possess a high PCE of 13.90% with a *J*_SC_ of 24.17 mA cm^−2^, an FF of 60.52%, and a *V*_OC_ of 0.950 V (shown in Figure S14 and Table S10, Supporting Information). The above results indicated that the PM6:PM6TPO:PY‐IT based ternary all‐PSCs is a promising approach for further industrial production.

In order to clarify the origin of the high *V*_oc_ delivered by PM6:PM6TPO:PY‐IT based all‐PSCs; the energy loss of the corresponding devices was analyzed according to Equation (1) ^[^ ^49^, ^50^ ^]^

(1) $$\begin{array}{ccc} E_{\mathrm{loss}} & = & E_{g}-qV_{\mathrm{OC}}\\ & = & \left(E_{g}-qV_{\mathrm{OC}}^{\mathrm{SQ}}\right)+\left(qV_{\mathrm{OC}}^{\mathrm{SQ}}-qV_{\mathrm{OC}}^{\mathrm{rad}}\right)+\left(qV_{\mathrm{OC}}^{\mathrm{rad}}-qV_{\mathrm{OC}}\right)\\ & = & \left(E_{g}-qV_{\mathrm{OC}}^{\mathrm{SQ}}\right)+qV_{\mathrm{OC}}^{\mathrm{rad},\mathrm{below}\;\mathrm{gap}}+qV_{\mathrm{OC}}^{\mathrm{non}-\mathrm{rad}}\\ & = & \Delta E_{1}+\Delta E_{2}+\Delta E_{3} \end{array}$$

First, *E*_g_ is the photovoltaic bandgap of the films can be estimated via the derivatives of the EQE spectra (d*E*Q*E*/d*E*),^[^ ^51^, ^52^ ^]^ which are shown in the Figure S15, Supporting Information. Δ*E*_1_ is the inevitable radiative loss of the BHJ devices, which can be calculated by the Shcoley–Queisser theory. The Δ*E*_1_ possess same value of 0.257 eV for all the binary and ternary devices. Δ*E*_2_ is the inevitable radiative loss below the bandgap due to the nonstep‐function‐like absorptance, exhibiting the values of 0.066, 0.065, and 0.064 eV for PM6, PM6TPO based binary devices and ternary all‐PSCs, respectively.

For the last part, Δ*E*_3_ is the nonradiative energy loss, which can be calculated according to Equation (2)

(2) $$\Delta E_{3}=-K_{B}T\left(\mathrm{In}\;EQE_{\mathrm{EL}}\right)$$

Where *K*_B_ is the Boltzmann constant, *T* is the absolute temperature, and *EQE*_EL_ is the external quantum efficiency of electroluminescence (EL), which are shown in Figure S16, Supporting Information. Δ*E*_3_ for these two binary devices based on PM6:PY‐IT and PM6TPO:PY‐IT are 0.196 and 0.200 eV, respectively. The PM6:PM6TPO:PY‐IT based ternary devices show relatively lower Δ*E*_3_ of 0.193 eV. Therefore, the total energy loss for all‐PSCs based on PM6:PY‐IT, PM6:PM6TPO:PY‐IT, and PM6TPO:PY‐IT are 0.519, 0.514, and 0.522 eV, respectively. All the detailed parameters are summarized in Table S11, Supporting Information.

## 3 Conclusion

In conclusion, we propose a novel “end‐capped” ternary strategy for highly efficient and stable all‐PSCs, on the basis of a set of “twins” polymer donor PM6TPO and PM6. This “end‐capped” assisted strategy can effectively improve device performance without additional selection and synthesis of the third component, which avoids complex third‐component selection and reduces costs of materials. The optimal ternary blends possess enhanced light harvesting and fine‐tuned morphology, the morphology study revealed the ternary blends exhibit the more suitable phase separation. Therefore, the all‐PSCs based on PM6:PM6TPO:PY‐IT achieves significantly enhanced *J*_SC_ of 24.80 mA cm^−2^, a higher FF of 72.52%, and resulting in an impressive PCE of 17.0%, which is one of the best performances among the all‐PSCs. This research demonstrates the importance of “end‐capped” ternary strategy for improving the photovoltaic performance and indicated the potential for further industrial application.

## Conflict of Interest

The authors declare no conflict of interest.

## Supporting information

Supporting Information

## Data Availability

The data that support the findings of this study are available from the corresponding author upon reasonable request.
