# Supplementary material for: All‐Polymer Solar Cells with 17% Efficiency Enabled by the “End‐Capped” Ternary Strategy
Source: Adv Sci (Weinh). 2022 Oct 3;9(32):2204030. doi: 10.1002/advs.202204030 (PMC9661854; doi:10.1002/advs.202204030)
Supplement: Supplementary file 1 — Supporting Information [file ADVS-9-2204030-s001.pdf]

ADVANCED SCIENCE
Open Access

Supporting Information

for *Adv. Sci.*, DOI 10.1002/advs.202204030

All-Polymer Solar Cells with 17% Efficiency Enabled by the "End-Capped" Ternary Strategy

Yuchen Yue, Bing Zheng, Jianling Ni, Wenjie Yang, Lijun Huo, Jingxia Wang* and Lei Jiang*

The All-Polymer Solar Cells with 17% Efficiency Enabled by the "end-capped" Ternary Strategy

Yuchen Yue, Bing Zheng, Jianling Ni, Wenjie Yang, Lijun Huo*, Jingxia Wang*, Lei Jiang

Abstract: Recently, all-polymer solar cells (all-PSCs) have received increasing attention and made tremendous progress. However, the power conversion efficiency (PCE) of all-PSCs still lags behind the polymer-donor-small-molecule-acceptor based organic solar cells, owing to the excessive phase separation with poor miscibility between polymer donor and acceptor. In this research, we propose a novel "end-capped" ternary strategy by introducing PM6TPO as a third component to fabricate highly efficient all-PSCs. The PM6:PM6TPO:PY-IT based ternary devices exhibit impressive PCE of 17.0% with enhanced light absorption and optimal morphology, and the introduction of PM6TPO significantly reduces the phase separation. The ternary devices also exhibit improved stability, outstanding tolerance of active layer thickness and high performance of 1 cm^2 unit cells. More importantly, the "end-capped" ternary strategy enables efficient and facile improvement of all-PSCs performance without additional selection and complicated synthesis for third component.

Experimental Procedures

1.Materials

PY-IT was purchased from Solarmer Materials Inc, PEDOT:PSS was purchased from Heraeus (Clevios Al 4083), PNDIT-F3N was purchased from eFlexPV Limited, Chloroform (anhydrous, 99.8%) was purchased from Sigma Aldrich, 1-CN was purchased from TCI. All materials were used directly after purchase, without any purification. PM6 and PM6TPO were synthesized by our group, follow our previous research.

The number average molecular weights (Mn) of PM6 and PM6TPO were determined to be 65.4 and 64.9 kDa with a polydispersity index (PDI) of 2.39 and 2.51, respectively, by gel permeation chromatography. Since the end-capped group possesses low molecular weight, PM6TPO as a product upon post-polymerization modification from PM6 exhibited a comparable Mn with PM6.

Table S1 The molecular weight of polymer donor

	Mn	*Mw*	PDI
PM6	65.4k	157.0k	2.39
PM6TPO	64.9k	162.3k	2.51

2.Measurements:

Optical measurements: The UV-visible spectrum was taken in UV-VIS-NIR spectrophotometer (AGILENT Cary 7000). Steady state photoluminescence (PL) spectra were acquired by a spectrometer (FLS1000).

Atomic force microscopy (AFM): AFM height and phase images were taken by Bruker Multimode 8. The samples for AFM were fabricated on ITO/PEDOT:PSS.

Transmission electron microscopy (TEM): TEM and DF-TEM images were recorded by using transmission electron microscope (HT-7700, JEOL 2100 and JEOL 2100F). The samples for TEM were fabricated on ITO/PEDOT:PSS.

Grazing incidence wide-angle X-ray scattering (GIWAXS) characterization: GIWAXS patterns were collect on XEUSS SAXS/WAXS system with incidence angle of 0.2º, the samples for GIWAXS were fabricated on silicon substrate.

Devices fabrication and testing:

The prepatterned ITO glasses were cleaned by detergent, deionized water, acetone, and isopropyl alcohol in ultrasonic bath for 20 minutes, respectively. After blow-drying by high-purity nitrogen， the ITO glasses were treated with oxygen plasma for 20 min at 200 W. A thin film of PEDOT:PSS was deposition on ITO by spin coating at 4200 rpm for 30s (the thickness is around 30 nm), and thermal annealed at 150°C for 15mins. Then the active layer was deposition by spin coating method in N_2-filled glove box. The active layer films were prepared from chloroform (CF) solvent with 0-2% 1-chloronaphthalene (1-CN) used as additive. The concentration of donor is from 6 to 8 mg/ml, the ratio of donor and acceptor was 1:1.2. The speed of spin coating was adjusted ranging from 3200 to 2000 mm/s to optimized. Subsequently, the active layers were heated at 100°C for 5 min. The methanol solution (contain 0.5% acetic acid) of PNDIT-F3N (0.5 mg/ml) was spin-coating on the active layer at 2200 rpm. Finally 100 nm Ag layer was thermally deposited under the vacuum condition of $1x10^{-4}$ Pa as top argentum electrode. The typical active area is 4.0 mm^2

Device current density-voltage (*J-V*) characteristics were recorded using the Keithley 2400 Source Measure Unit. The photocurrent was tested under AM 1.5G illumination at 100 $mWcm^{-2}$ by a solar simulator (SCISUN, Ligntsky Technology Co). The forward scan was adopted to test the J-V curves, the scan step is 0.02 V and the delay time is 1ms. The scan mode is sweep. The light intensity was calibrated by standard Si solar cell (LRC-KG2, Ligntsky Technology Co). The EQE spectra were measured by using a solar-cell spectral-response measurement system (QE-R3011, Enlitech).

Space-charge-limited-current (SCLC) mobility measurement: Single carrier devices were fabricated, and the dark *J-V* characteristics measured in the space charge limited (SCLC)regime following the references. The structure of hole only devices was ITO/PEDOT:PSS/active layer/MOo3(5 nm)/Ag(100nm). For the electron only devices, the structure was ITO/ZnO/active layer/PNDIT-F3N/Ag (100 nm) where the Ag were evaporated. The J-V characteristics of both hole-only and electron-only devices can fit to SCLC relation: $J=9\varepsilon_0\varepsilon_r\mu V^2/8d^3$.where J is the current density, d is the film thickness of the active layer, μ is the charge carrier mobility, ε_r is the relative dielectric constant of the transport medium, and ε_0 is the permittivity of free space.

PL and TRPL spectra

Steady state photoluminescence (PL) spectra are obtained by FLS1000. Fluorescence decay dynamics are acquired by TCSPC. The excitation wavelength is 400 nm, with laser power of 76 μW.

Femtosecond transient absorption spectrum:

A Ti: sapphire amplifier (Coherent Co.) supplied laser beams centered at 800 nm with pulse duration of 25 fs, pulse repetition rate of 1 KHz, and a maximum pulse energy of 4 mJ. The output of the amplifier was split into two streams of pulses with a beam splitter. Residual stream was directed into an ultrafast spectroscopic system (Helios pump-probe system (Ultrafast Systems) to generate the white light continuum probe beam. The film samples for TA measurements are prepared on the quatz plates. The TA samples are annealed in nitrogen glove box at 100°C for 5 min.

Table S2 The summarized optical properties and energy level of materials.

Materials	λ^{sol}_{max} (nm)	λ^{film}_{max} (nm)	λ^{film}_{onset} (nm)	E^{g}_{opt} (e*V*)	HOMO (e*V*)	LUMO (e*V*)	E^{g}_{CV} (e*V*)
PM6	577	577	695	1.86	-5.52	-3.62	1.9
PM6TPO	577	577	665	1.78	-5.51	-3.61	1.9
PY-IT	795	820	895	1.38	-5.60	-3.97	1.63

Table S3 The summarized parameters of devices photovoltaic parameters with different weight ratio.

J_{SC} (mA/cm^2)	V_{OC} (V)	FF (%)	PCE (%）	PM6:PM6TPO:PY-IT
22.96[a)] (22.70±0.30)[b)]	0.940 [a)] (0.935±0.05)[b)]	71.77[a)] (71.20±0.50) [b)]	15.49 [a)]/(15.22±0.25) [b)]	1:0:1
23.20 [a)] (23.00±0.20)[b)]	0.941 [a)] (0.935±0.05)[b)]	71.16 [a)] (70.80±0.40) [b)]	15.65 [a)]/(15.30±0.35) [b)]	0.9:0.1:1
24.22 [a)] (24.00±0.20)[b)]	0.945 [a)] (0.940±0.05)[b)]	70.60 [a)] (70.00±0.60) [b)]	16.18 [a)]/(15.80±0.40) [b)]	0.7:0.3:1
24.80 [a)] (24.60±0.20)[b)]	0.945 [a)] (0.940±0.05)[b)]	72.52 [a)] (72.00±0.50) [b)]	17.0 [a)]/(16.73±0.30) [b)]	0.5:0.5:1
24.68 [a)] (24.40±0.30)[b)]	0.943 [a)] (0.940±0.05)[b)]	70.10 [a)] (69.80±0.30) [b)]	16.32 [a)]/(16.00±0.30) [b)]	0.3:0.7:1
23.90 [a)] (23.60±0.30)[b)]	0.938 [a)] (0.933±0.05)[b)]	69.31 [a)] (69.00±0.30) [b)]	15.54 [a)]/(15.22±0.35) [b)]	0.1:0.9:1
23.43 [a)] (23.00±0.40)[b)]	0.937 [a)] (0.932±0.05)[b)]	69.50 [a)] (69.20±0.30) [b)]	15.26 [a)]/(15.03±0.23) [b)]	0:1:1

a) The best performance of devices.
b) Values in brackets are average based on more than 30 independent devices.

Table S4 The charge mobility of the active layer.

Active Layer	μ_h ($cm^2V^{-1}s^{-1}$)	μ_e ($cm^2V^{-1}s^{-1}$)	μ_h:μ_e
PM6	$1.34*10^{-3}$	-	-
PM6TPO	$1.23*10^{-3}$	-	-
PY-IT	-	$1.10*10^{-3}$	-
PM6:PY-IT	$7.6*10^{-4}$	$5.5*10^{-4}$	1.38:1
PM6TPO:PY-IT	$6.9*10^{-4}$	$5.4*10^{-4}$	1.27:1
PM6:PM6TPO:PY-IT	$8.5*10^{-4}$	$7.0*10^{-4}$	1.21:1

Table S5 The summarized parameters of optimal binary and ternary devices.

Active layer	J_{ph} (mA/cm^2)	J_{max} (mA/cm^2)	J_{sat} (mA/cm^2)	η_{diss}	η_{coll}
PM6:PYIT	22.35	18.66	23.21	96.29	80.39
PM6TPO:PYIT	22.28	18.78	23.28	95.70	80.67
PM6:PM6TPO:PYIT	24.56	21.54	25.17	97.58	87.70

Table S6 The summarized crystalline parameters of neat and blend film.

Material	(100) IP				(010) OOP			
	q/ Å^{-1}	d/ Å	FWHM	CCL/ Å	q/ Å^{-1}	d/ Å	FWHM	CCL/ Å
PM6	0.293	21.44	0.112	50.48	1.66	3.79	0.266	21.26
PM6:PM6TPO	0.293	21.44	0.117	48.33	1.664	3.776	0.283	19.98
PM6TPO	0.293	21.44	0.123	45.97	1.668	3.767	0.300	18.84
PY-IT	0.370	17.00	0.153	37.00	1.636	3.84	0.251	22.50
PM6:PY-IT	0.297	21.15	0.068	83.15	1.644	3.82	0.3347	16.90
PM6:PM6TPO:PY-IT	0.295	21.29	0.072	78.54	1.652	3.8	0.3366	16.80
PM6TPO:PY-IT	0.295	21.29	0.0844	67	1.654	3.798	0.3386	16.70

Surface Tension Calculation: The contact angles of two different solvent (water and Glycerol) on neat films of polymer donor PM6, two small molecular donors Z1, Z2, and Y6 were used to calculate the surface tension by Wu model. The detailed calculation process is described below：

$$\gamma_{\mathrm{Water}}\,(\cos\theta+1)=\frac{4\gamma_{Water}^{d}\gamma^{d}}{\gamma_{Water}^{d}+\gamma^{d}}+\frac{4\gamma_{Water}^{p}\gamma^{p}}{\gamma_{Water}^{p}+\gamma^{p}}$$

$$\gamma_{\mathrm{Glycerol}}\,(\cos\theta+1)=\frac{4\gamma_{\mathrm{Glycerol}}^{d}\gamma^{d}}{\gamma_{\mathrm{Glycerol}}^{d}+\gamma^{d}}+\frac{4\gamma_{\mathrm{Glycerol}}^{p}\gamma^{p}}{\gamma_{\mathrm{Glycerol}}^{p}+\gamma^{p}}$$

$$\gamma=\gamma^{d}+\gamma^{p}$$

Where θ is the contact angle of films and γ is the surface tension of the films which is the sum of dispersion (γ^d) and polarity (γ^p) components. The γ_{Water} and $\gamma_{Glycerol}$ are the surface tensions of the water and glycerol.

The Compatibility between different materials can be deduced from the empirical formula of Flory-Huggins parameter.

$$\chi \propto \left(\sqrt{\gamma_A}\text{-}\sqrt{\gamma_B}\right)^2$$

Table S7 Summarized parameters for the surface tension of the films.

Materials	PM6	PM6TPO	PY-IT
γ/mN*m^{-1}	19.82	20.25	23.11

Table S8 Summarized Flory-Huggins parameter for the different materials.

	PM6TPO: PM6	PM6: PY-IT	PM6TPO: PY-IT
$\chi \propto \left(\sqrt{\gamma_A}\text{-}\sqrt{\gamma_B}\right)^2$	0.0023	0.1262	0.0944

Table S9 The summarized parameters of devices photovoltaic parameters with different active layer thickness.

Jsc (mA/cm^2)	Voc (V)	FF (%)	PCE (%)	Thickness (nm)
24.20 [a)] (22.70±0.30)[b)]	0.934 [a)] (0.930±0.05)[b)]	64.56 [a)] (64.50±0.50) [b)]	14.62 [a)]/(14.13±0.50) [b)]	200
24.23 [a)] (22.70±0.30)[b)]	0.942 [a)] (0.935±0.05)[b)]	69.22 [a)] (68.90±0.30) [b)]	15.85 [a)]/(15.25±0.60) [b)]	180
24.8 [a)] (22.70±0.30)[b)]	0.945 [a)] (0.940±0.05)[b)]	69.91 [a)] (69.70±0.20) [b)]	16.37 [a)]/(16.00±0.40) [b)]	160
24.8 [a)] (22.70±0.30)[b)]	0.945 [a)] (0.940±0.05)[b)]	72.52 [a)] (72.30±0.20) [b)]	17.00 [a)]/(16.73±0.30) [b)]	140
24.22 [a)] (22.70±0.30)[b)]	0.946 [a)] (0.940±0.05)[b)]	71.02 [a)] (70.50±0.50) [b)]	16.42 [a)]/(16.10±0.30) [b)]	120
24.02 [a)] (22.70±0.30)[b)]	0.948 [a)] (0.943±0.05)[b)]	71.55 [a)] (71.20±0.30) [b)]	16.30 [a)]/(16.13±0.20) [b)]	100
23.60 [a)] (22.70±0.30)[b)]	0.950 [a)] (0.945±0.05)[b)]	73.00 [a)] (72.50±0.50) [b)]	16.40 [a)]/(16.00±0.40) [b)]	80

a) The best performance of devices.
b) Values in brackets are average based on more than 30 independent devices.

Table S10 The summarized parameters of 1 cm^2 devices photovoltaic parameters based on PM6:PM6TPO:PY-IT blend.

Jsc (mA/cm^2)	Voc (V)	FF (%)	PCE (%)	AREA
24.17 [a)]/23.45 [b)] (23.90±0.30)[c)]	0.950 [a)] (0.945±0.05)[c)]	60.56 [a)] (60.00±0.50)[c)]	13.90 [a)] (13.60±0.030)[c)]	1 cm^2

a) The best performance of devices.
b) The integrated J_{sc} values from EQE spectra.
c) Values in brackets are average based on more than 30 independent devices.

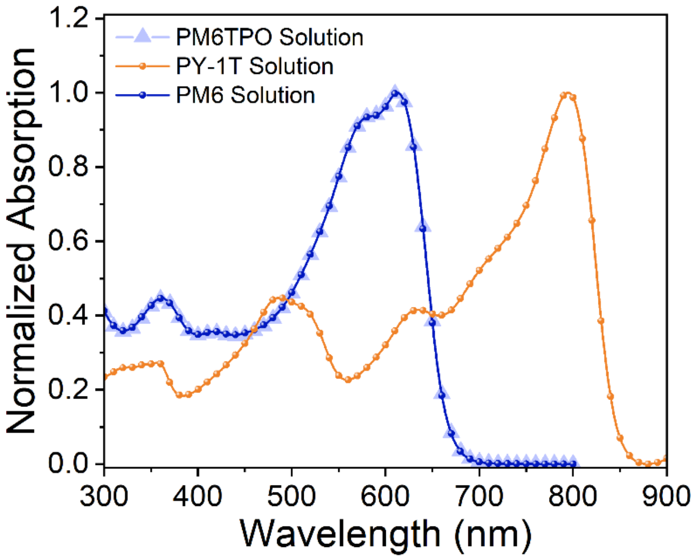

Figure S1 The UV-vis absorption spectra of materials in solution.

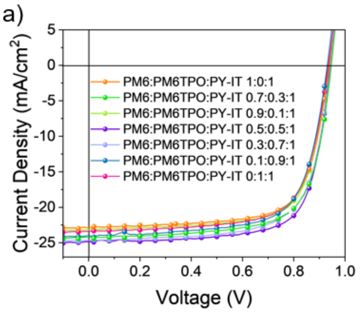

Figure S2 a) the *J-V* curves of ternary devices with different weight ratio.

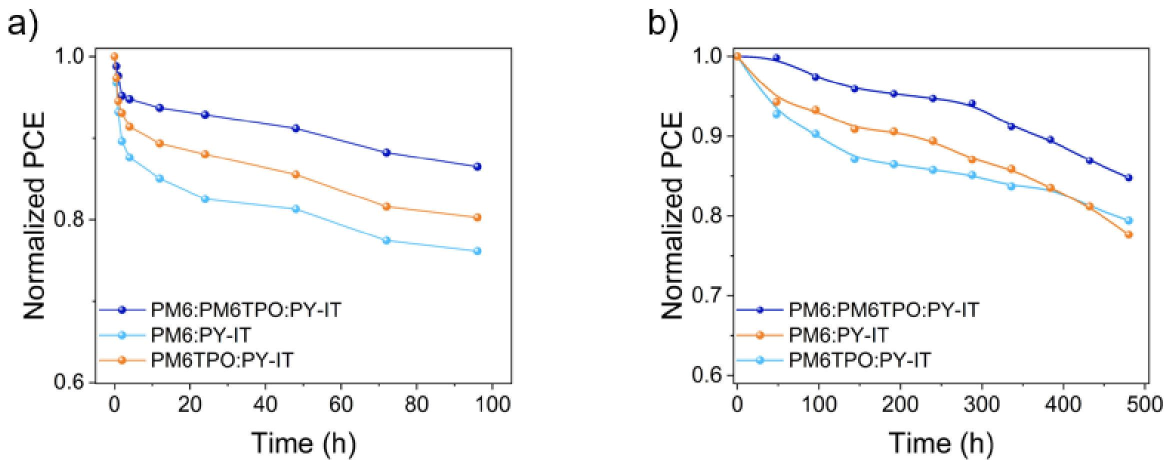

Figure S3 a) The thermal stability for ternary and binary devices, which are heated at 100°C. b) The photo stability for ternary and binary devices under illumination at 100 $mWcm^{-2}$ by a LED simulator.

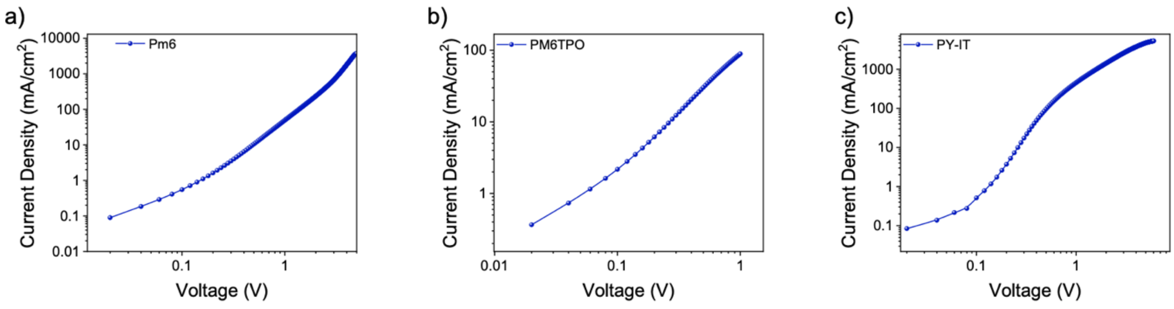

Figure S4 The charge mobility of a) PM6, b) PM6TPO and c) PY-IT.

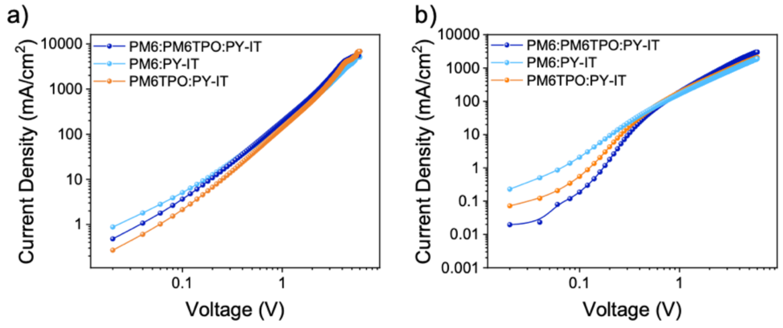

Figure S5 The charge mobility of binary and ternary devices with a) hole-only devices and b) electron-only devices, measured by SCLC method.

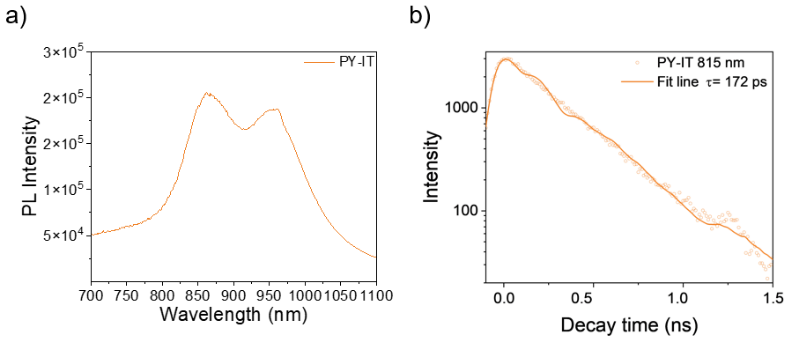

Figure S6 The a) PL spectra of PY-IT, b) the TRPL spectra of PY-IT.

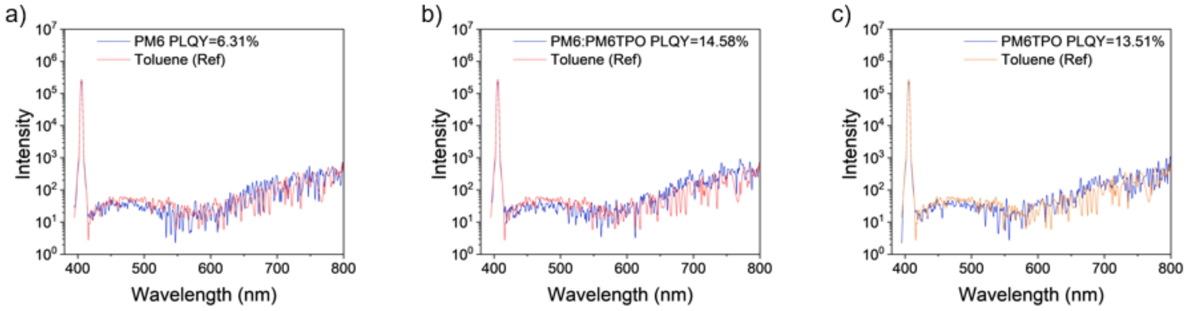

Figure S7 The PL quantum yield (PLQY) of a) PM6, b) PM6:PM6TPO and c) PM6TPO. The PM6 and PM6TPO neat films, and their binary film exhibited PLQY of 6.31%, 13.51% and 14.58%, respectively.

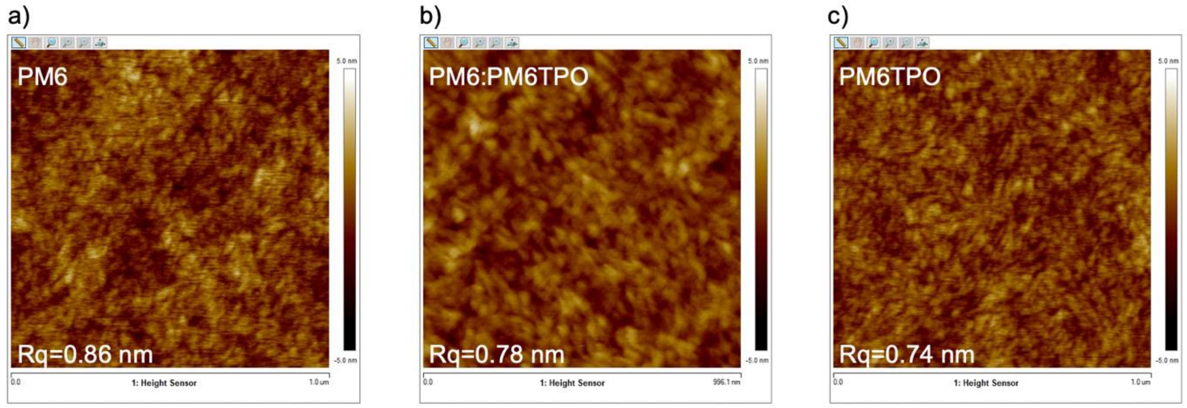

Figure S8 The AFM height images of a) PM6, b) PM6:PM6TPO and c) PM6TPO.

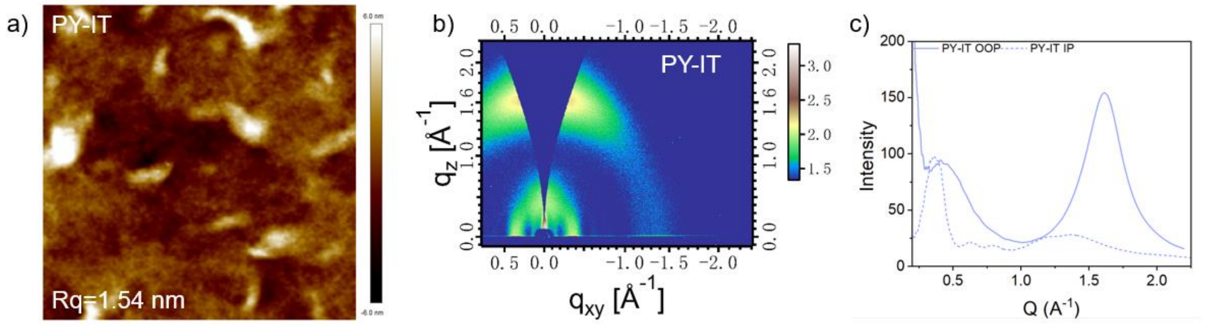

Figure S9 The measurement of the PY-IT pristine films. a) AFM height images of the PY-IT. b) GIWAXS patterns of the PY-IT film. c) OOP Line cuts for corresponding GIWAXS patterns of PY-IT.

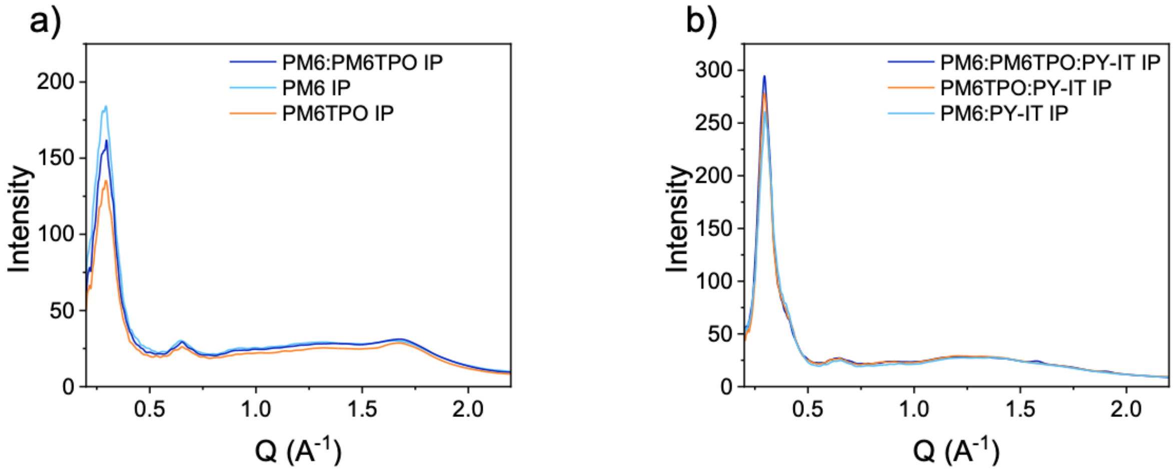

Figure S10 The line cuts of a) neat films and b) blend films in the IP direction.

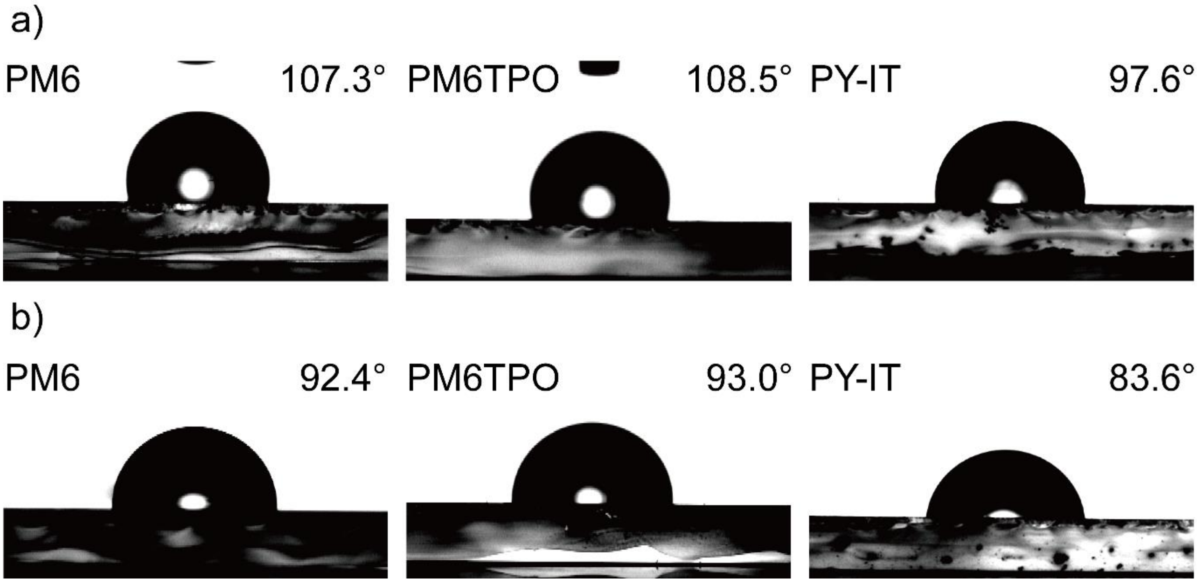

Figure S11. Contact angle of PM6, PM6TPO and PY-IT neat films for water a) and glycerol b).

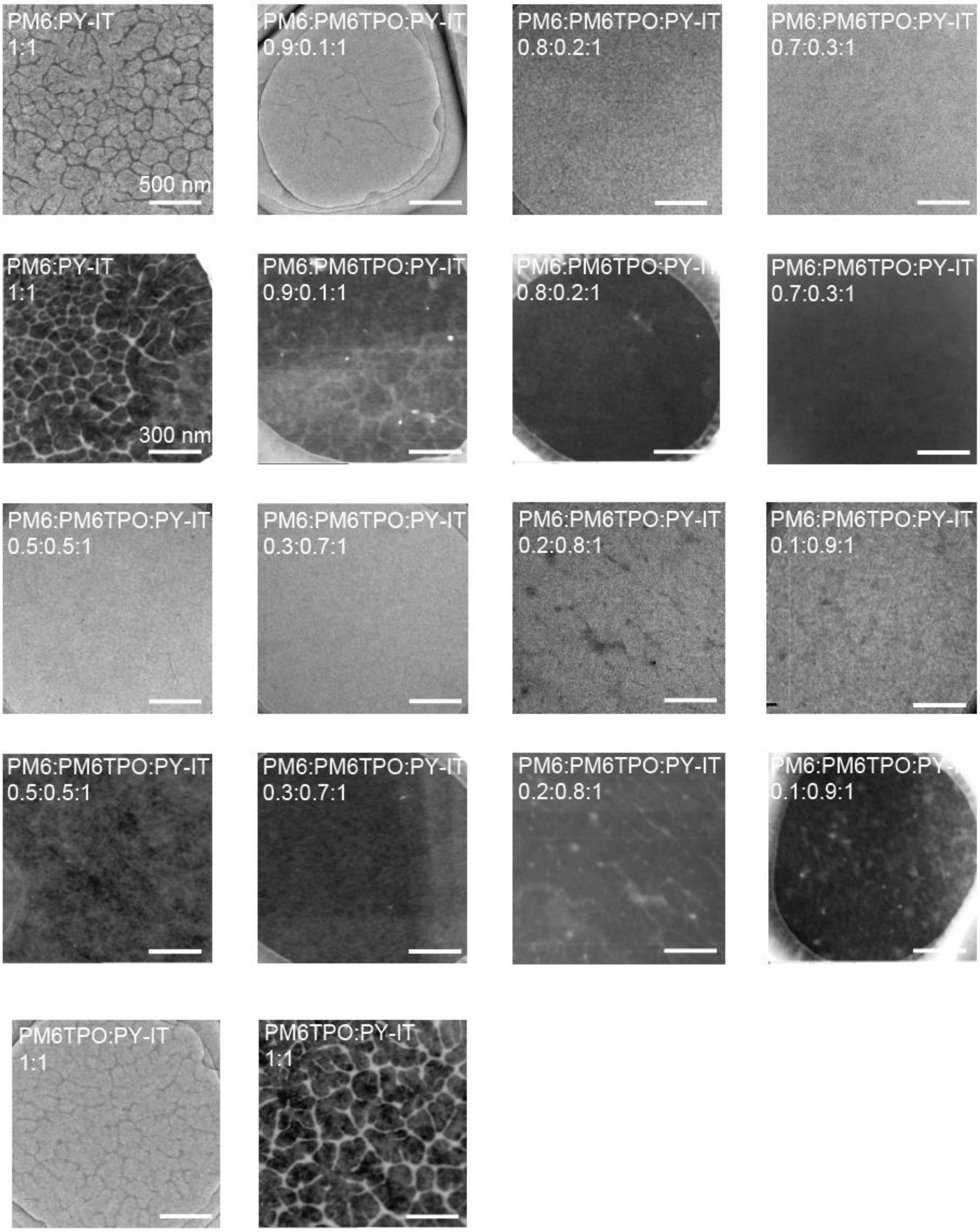

Figure S12 The complete morphology evolution process from binary systems to ternary systems.

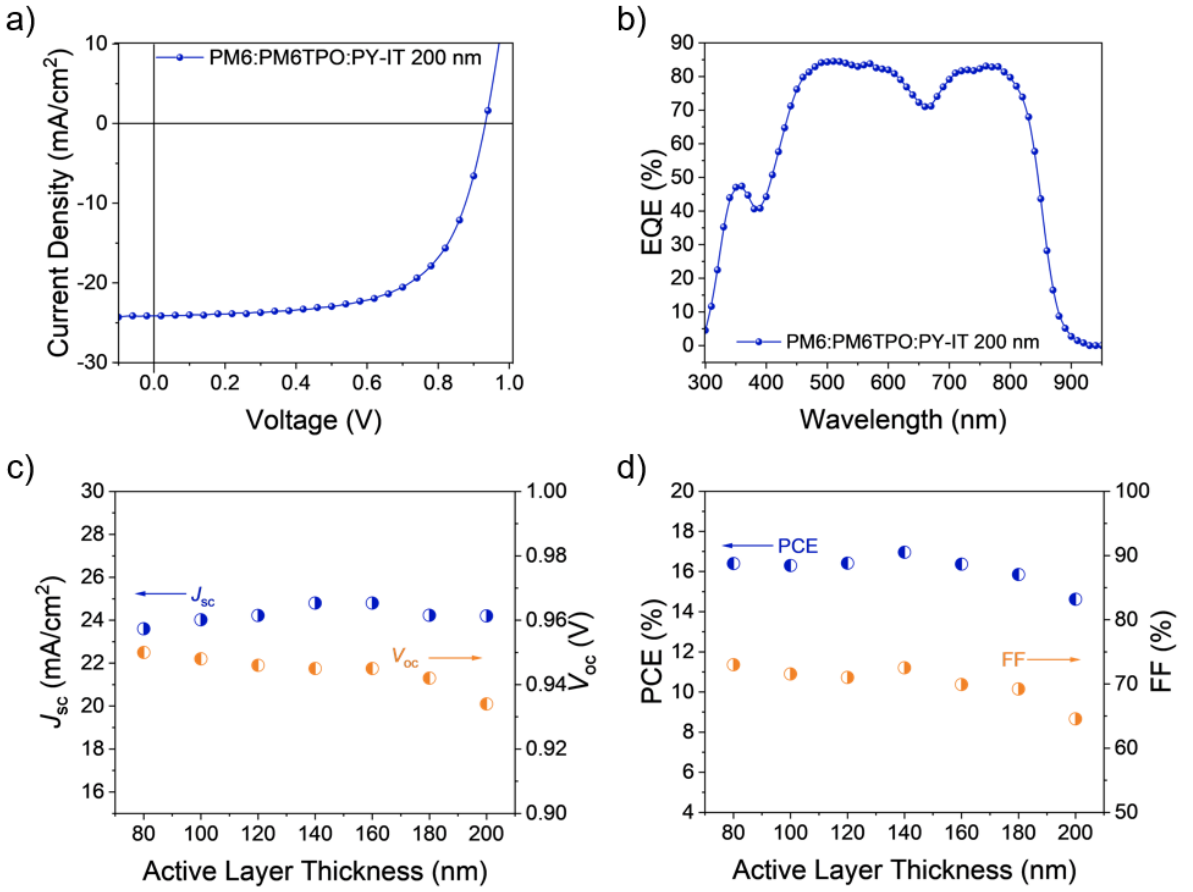

Figure S13 a) The *J-V* curve and b) EQE spectra of ternary devices of 200 nm active layer. c) The plot of the J_{SC} and V_{OC} versus the active layer thickness ranging from 80 to 200 nm; d) The plot of the PCE and FF versus the active layer thickness ranging from 80 to 200 nm.

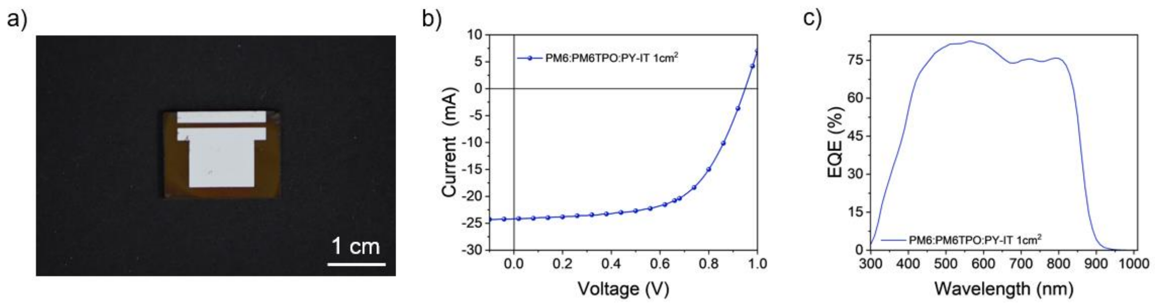

Figure S14 a) The photograph of 1cm^2 unit cell based on the PM6:PM6TPO:PY-IT; b) The *J*-*V* curve and c) EQE spectra of 1cm^2 unit cell.

Energy loss characterzition

The (Electroluminescence) EL spectra were taken using a Kymera-328I spectrograph and an EMCCD purchased from Andor Technology (DU970P). Injection current used for EL was 1 mA cm $^{-2}$. EQEEL measurements were done using a home-built setup using a Keithley 2400 to inject current to the solar cells. Emission photon-flux from the solar cells was recorded using a Si detector (Hamamatsu s1337-1010BQ) and a Keithley 6482 picoammeter.

Details of optical-gap determination

As previously reported in the literature, an EQE is interpreted as a superposition of a distribution of step-functions with a step at *Eg* having a certain probability distribution. This probability distribution can be obtained from the derivative dEQE/dE. The part where the probability is greater than half of the maximum is integrated to get an average gap.

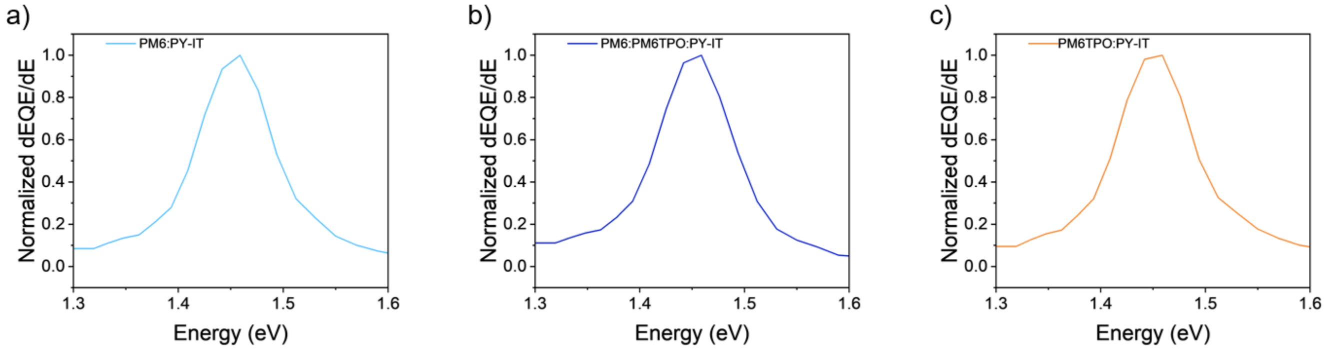

Figure S15.Determination of the *Eg* of the binary and ternary devices via the derivatives of the EQE spectra.

The value of V_{OC}^{SQ} can be caiculated according to the following equation:

$$V_{OC}^{SQ} = \frac{K_B T}{q} \text{In}(\frac{J_{SC}^{SQ}}{J_0^{SQ}} + 1) \cong \frac{K_B T}{q} \text{In}(\frac{q \times \int_{Eg}^{+\infty} \emptyset_{AM1.5G}(E) \times \mathrm{d}E}{q \times \int_{Eg}^{+\infty} \emptyset_{BB}(E) \times \mathrm{d}E})$$

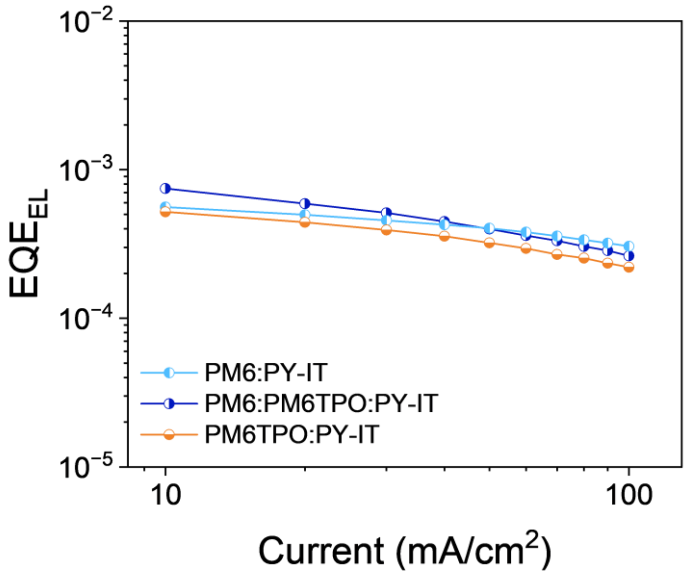

Figure S16 EQE_{EL} of PM6:PY-IT,PM6:PM6TPO:PY-IT and PM6TPO:PY-IT based all-PSCs.

Table S11 Detailed energy loss of these binary and ternary devices.

Devices	E_g (eV)	V_{oc} (V)	V_{loss} (V)	V^{SQ}_{OC}(V)	E_1(eV)	E_2(eV)	E_3(eV)	EQE_{EL}
PM6:PY-IT	1.459	0.94	0.519	1.202	0.257	0.066	0.196	4.85*10-4
PM6:PM6TPO:PY-IT	1.459	0.945	0.514	1.202	0.257	0.064	0.193	5.50*10-4
PM6TPO:PY-IT	1.459	0.938	0.522	1.202	0.257	0.065	0.200	4.24*10-4

Author Contributions

Jingxia Wang and Lijun Huo proposed the research direction and guided the project; Yuchen Yue designed and performed the experiments and drafted the original manuscript. Bing Zheng helped with the material synthesis. Jianling Ni helped the supplementary fabrication of devices. Wenjie Yang helped the TEM measurement. Lei Jiang conceptualized the manuscript and administrated the project. Jingxia Wang and Lijun Huo revised the manuscript. All authors contributed to the writing of the paper.
